# Vascular Endothelial Glycocalyx Damage and Potential Targeted Therapy in COVID-19

**DOI:** 10.3390/cells11121972

**Published:** 2022-06-19

**Authors:** Duoduo Zha, Mingui Fu, Yisong Qian

**Affiliations:** 1 The National Engineering Research Center for Bioengineering Drugs and the Technologies, Institute of Translational Medicine, Nanchang University, Nanchang 330031, China; zdd20170907yx@163.com; 2 Shock/Trauma Research Center, Department of Biomedical Sciences, School of Medicine, University of Missouri Kansas City, Kansas City, MO 64108, USA; fum@umkc.edu

**Keywords:** COVID-19, endothelial cells, glycocalyx, syndecan-1, heparanase

## Abstract

COVID-19 is a highly infectious respiratory disease caused by a new coronavirus known as SARS-CoV-2. COVID-19 is characterized by progressive respiratory failure resulting from diffuse alveolar damage, inflammatory infiltrates, endotheliitis, and pulmonary and systemic coagulopathy forming obstructive microthrombi with multi-organ dysfunction, indicating that endothelial cells (ECs) play a central role in the pathogenesis of COVID-19. The glycocalyx is defined as a complex gel-like layer of glycosylated lipid–protein mixtures, which surrounds all living cells and acts as a buffer between the cell and the extracellular matrix. The endothelial glycocalyx layer (EGL) plays an important role in vascular homeostasis via regulating vascular permeability, cell adhesion, mechanosensing for hemodynamic shear stresses, and antithrombotic and anti-inflammatory functions. Here, we review the new findings that described EGL damage in ARDS, coagulopathy, and the multisystem inflammatory disease associated with COVID-19. Mechanistically, the inflammatory mediators, reactive oxygen species (ROS), matrix metalloproteases (MMPs), the glycocalyx fragments, and the viral proteins may contribute to endothelial glycocalyx damage in COVID-19. In addition, the potential therapeutic strategies targeting the EGL for the treatment of severe COVID-19 are summarized and discussed.

## 1. Introduction

The emergence of severe acute respiratory syndrome coronavirus 2 (SARS-CoV-2), which causes the coronavirus disease 2019 (COVID-19), triggered a global pandemic that has led to an unprecedented worldwide public health crisis. Early in COVID-19, SARS-CoV-2 enters pulmonary epithelial cells via surface angiotensin-converting enzyme 2 (ACE2) receptors, resulting in viral pneumonia, followed by a systemic inflammatory phase [1,2,3]. Typical clinical symptoms of SARS-CoV-2 infection includes acute respiratory distress syndrome (ARDS), systemic inflammatory response syndrome (SIRS), multiple organ failure (MOF), and disseminated intravascular coagulation (DIC) [2,4,5]. Some other clinical events usually observed in COVID-19 patients, including high blood pressure, thrombosis kidney disease, pulmonary embolism, Kawasaki disease, cerebrovascular and neurologic disorders, mesenteric ischemia, and cutaneous vasculitis, all indicating that the virus impairs vascular endothelial function [6,7]. Clinical markers for indicating the activation of coagulation and fibrinolysis, including d-dimer and Von Willebrand factor (VWF), were significantly elevated in COVID-19 patients and were predictive of poor outcome, further supporting the hypothesis of SARS-CoV-2-induced endothelial damage [8,9].

Extensive endothelial dysfunction during COVID-19 is considered as a consequence of a cytokine storm of hyper-inflammation [9]. IL-6 caused endothelial activation and neutrophil infiltration, which resulted in changes to vascular permeability and loss of vascular tone and increased microvascular complications [9]. The pro-inflammatory cytokine TNF-α-induced release of NOX also contributed to local oxidative stress and endothelial dysfunction during COVID-19 [10]. Endothelial activation, characterized by the increased production of adhesion molecules, is an early hallmark of multiple organ failure in patients with COVID-19 [11]. The inflammatory cell infiltration around blood vessels and ECs, microvascular thrombosis and angiogenesis, endothelial glycocalyx damage, and other clinical manifestations related to endothelial injury were observed in patients with severe COVID-19 [12,13,14], and thrombotic complications have been linked to mortality in COVID-19 patients [15]. Pathological findings of cell swelling, severe endothelial injury, disruption of intercellular junctions, and basal membrane contact loss in COVID-19 patients imply that the destruction of ECs leads to pulmonary vascular endotheliitis and alveolar capillary microthrombi [12,14]. Qin et al. revealed that SARS-CoV-2 caused endotheliitis via both infection and infection-mediated immune activation in animal models as well as in severe COVID-19 patients [16]. Several potential therapeutic targets associated with ECs for defeating COVID-19, including ACE2 [17], transmembrane protease serine 2 (TMPRSS2) [18,19], chemokine receptor 5 (CCR5) [19], IL-8 [19], CXCL-8 receptor (CXCR-2) [19], nitric oxide (NO) [20], and Neuropilin-1 [21,22]. The development of materials science also provided innovative technologies including 3D-printed models, engineered endothelium, and nanosized delivery systems for understanding the molecular mechanisms underlying endothelial dysfunction in COVID-19 patients [23].

The endothelial glycocalyx layer (EGL) plays an essential role in vascular homeostasis and the EGL damage is closely associated with vascular endothelial dysfunction [24,25]. A number of studies show that the EGL is damaged in severe COVID-19 patients and the increased plasma levels of glycocalyx components, such as syndecan-1, heparan sulphate, and hyaluronan, were observed as biomarkers, accompanied with high levels of IL-1β, IL-6, TNF-α, hsCRP, and procalcitonin, increasing the risk for COVID-19 severity and mortality [26,27,28,29]. Additionally, several drugs that protect EGL from damage are already used in COVID-19, such as heparin and tocilizumab [26,30,31,32]. However, though there have been many reports of glycocalyx injury in COVID-19, its mechanism remains to be explored. Here, we reviewed the recent findings of the EGL damage in severe COVID-19. In addition, the mechanisms of COVID-19-induced endothelial glycocalyx damage and the potential therapeutic strategies were also summarized and discussed.

## 2. Endothelial Glycocalyx in Vascular Hemostasis

### 2.1. Endothelial Glycocalyx Components

**Figure 1:**
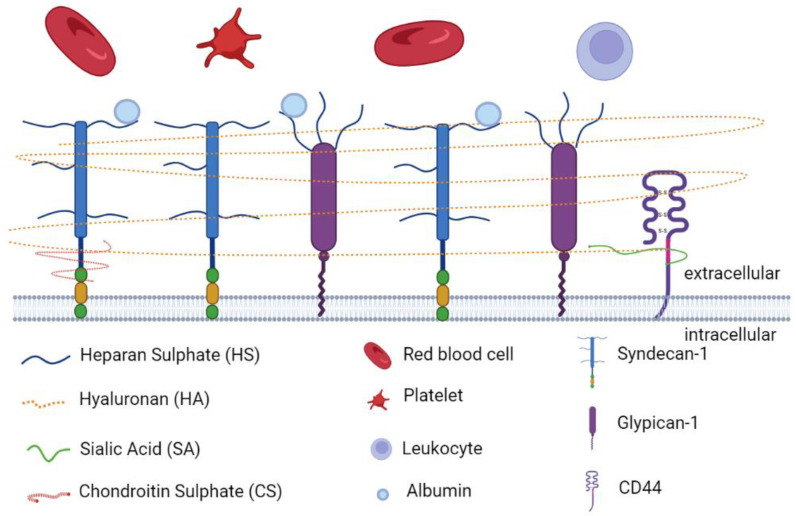
The main components of endothelial glycocalyx. The endothelial glycocalyx layer is a glycoprotein network comprising membrane-binding proteoglycans with glycosaminoglycan side chains (HS, HA, SA, and CS), glycoproteins (syndecan-1 and glypican-1) bearing acidic oligosaccharides and terminal sialic acids, plasma proteins (albumin), and receptors (CD44). The figure was created by the online software tool BioRender.

Glycocalyx is defined as a complex gel-like layer of glycosylated lipid–protein mixtures, which surround all living cells and serves as a buffer between cells and the extracellular matrix (ECM) [25,33,34]. The glycocalyx layer is uniform in leucocytes, neurons, fibrocytes, and other cells of mesenchymal origin, whereas it appears thicker at apical than at lateral and basal membranes in epithelial cells [34,35]. Endothelial glycocalyx layer (EGL) is synthesized and secreted by vascular ECs and extends along the endothelial layer covering the luminal surface of blood vessels [36]. As is shown in Figure 1, EGL is a glycoprotein network comprising membrane-binding proteoglycans with glycosaminoglycan side chains, glycoproteins bearing acidic oligosaccharides and terminal sialic acids, and plasma proteins (such as albumin and antithrombin) [37,38]. The syndecan and glypican families are the major proteoglycans tightly bound to the EC membrane [39,40]. There are five main components of glycosaminoglycan (GAG) side chains: heparan sulphate (HS), chondroitin sulphate (CS), dermatan sulphate (DS), keratan sulphate (KS), and hyaluronan (HA). Among them, HS is the most abundant component in GAG side chains, accounting for 50% to 90% of these chains [38]. Under normal physiological conditions, GAG chains are highly sulphated, presenting a negatively charged nature [41], which facilitates its interaction with plasma constituents [40]. The charged glycocalyx functions as a macromolecular sieve and negatively repels white blood cells, red blood cells, platelets, and other charged molecules [39,42,43]. Different from other GAG chains, HA is an uncharged, non-sulphated GAG chain, accounting for 5% to 20% of the total GAG chains of the EGL [44]. HA can form complexes with other sulphated GAG chains to sequester water and stabilize the network of the glycocalyx [45]. A nonkinase cell surface receptor, CD44, serves as the major membrane receptor for HA [44].

The EGL varies widely in thickness and structure. This difference may be caused by species, organs, vascular beds, and blood flow velocities [44,46]. Therefore, relative changes in glycocalyx thickness or composition should be addressed rather than focusing on their absolute values [44].

### 2.2. Physiological Roles of EGL in Vascular Homeostasis

EGL plays an important role in vascular homeostasis. It regulates vascular permeability and cell adhesion, acts as a mechanosensor for hemodynamic shear stresses, and exerts antithrombotic and anti-inflammatory functions [43]. In the physiological condition, EGL plays a key role in maintaining the transvascular exchange of water and solutes [24,47,48] and acts as the barrier that prevents albumin and other circulating plasma components from passing through the ECs [43,49]. HA and HS are mainly responsible for the selective permeability of ECs [44]. In addition, albumin usually has a protective effect against the degradation of glycocalyx, which helps to maintain the vascular integrity and normal permeability [43,50]. Sialic acid (SA) is one of the important components of EGL that caps a range of EGL glycoproteins [37,51,52]. Henry [37] et al. proved that sialidases-mediated disruption of EGL could induce hyperpermeability in human pulmonary microvascular ECs. Betteridge [52] et al. also demonstrated that SA residues within EGL are principal regulators of microvascular permeability.

Leukocyte adhesion is a critical step in the development of immune and inflammatory responses [53]. Endothelial selectins interact with the leukocyte surface ligands and mediate the rolling of neutrophils, monocytes, and T lymphocytes on ECs [54], promoting the binding of integrins on the leukocyte surface to the immunoglobulin superfamily molecules (such as ICAM-1 and VCAM-1) on the endothelial surface. Subsequently, the leukocyte cytoskeleton undergoes remodeling and facilitates tight adherence to ECs [55,56,57]. Under physiological conditions, the adhesion molecules and their receptors are embedded in the EGL to prevent the binding of the leukocytes [58,59]. In inflammatory conditions, pro-inflammatory cytokines and mediators induce the shedding of the glycocalyx, exposing adhesion molecules and initiating leukocyte adhesion [60]. The neutrophil/monocyte-EC interaction allows leukocyte infiltration into surrounding tissue to destroy pathogens, but the activated macrophages, monocytes, and other cells also mediate local responses leading to tissue damage and organ failure [56].

The vascular ECs are continuously exposed to the mechanical forces generated by blood flow. When EGL is intact, shear stress can be transmitted to the actin cytoskeleton or directly to cell membrane through glycoprotein, thereby mediating cell signal transduction [61]. Studies have found that glypican-1 transmits the fluid share force sensed by the HS chains to the signaling machinery and also play a role in shear-induced nitric oxide (NO) production [62,63,64]. Heparanase, responsible for HS degradation, can inhibit mechanical forces-induced NO production, resulting in vasomotor dysfunction [51]. The GAG-cleaving enzymes also cause EGL damage and disrupt mechanosensing, resulting in a marked reduction in NO production [64,65]. 

EGL also maintains the delicate balance between blood coagulation and anticoagulation [61]. EGL affect the coagulation cascade through the interaction with tissue factor pathway inhibitor (TFPI), an effective inhibitor of FVIIa and Fxa in the coagulation pathway [40]. HS binds to circulating antithrombin III to enhance its anticoagulant action [61,66] and CS binds to thrombomodulin forming the anticoagulation pathway [40]. Moreover, when glycocalyx is shed, HS and CS with anticoagulant activity can be released into the blood circulation, causing autoheparinization and coagulopathy [67].

### 2.3. Regulation of the EGL Degradation

Plasma levels of the EGL components such as HS, HA, and syndecan-1, indicating the EGL shedding, were increased during viral or bacterial infections [25]. It has been reported that the plasma levels of syndecan-1 and HA significantly increased in COVID-19 patients [68], and the degradation of the EGL would deteriorate the conditions of COVID-19 patients [3,25]. The EGL degradation is mediated by the enzymatic and nonenzymatic manners. The enzymatic EGL degradation is usually induced by the EGL sheddase such as heparanase, matrix metalloproteases (MMPs), and hyaluronidases. Heparanase is the only known mammalian enzyme capable of cleaving HS [69], which is the most abundant GAG and the main contributor to the negative-charge-dependent barrier [38,70,71]. Heparanase is synthesized in the endoplasmic reticulum and undergoes the cleavage of a linker region by cathepsin L, forming the mature heparanase [70]. HS has a central role in the inflammatory response by controlling the release of pro-inflammatory cytokines, regulating the leukocytes–EC interaction, and facilitating leukocyte recruitment, rolling, and extravasation [72,73]. Therefore, the elevated heparanase levels play a significant role in inflammation-mediated damage of EGL and are implicated in a variety of diseases [74]. The HS chains of syndecan-1 are also shed by MMPs, such as MMP-3 and MMP-7. The shedding of HS and syndecan-1 is not independent from each other. Upon the cleavage of HS by heparanase, the ectodomain of syndecan-1 is exposed and cleaved by MMPs [69]. Hyaluronidases, a family of enzymes that catalyze the degradation of HA, are responsible for the regulation of glycocalyx thickness and hence the access of circulating cells and factors to the endothelial cell membrane [75]. The altered oxidation–reduction state and ensuing microinflammation were considered directly associated with the activation of hyaluronidases and loss of the glycocalyx [75].

The nonenzymatic EGL degradation often appears in pathologic conditions. ROS and myeloperoxidase (MPO) were found to be involved in the cleavage of syndecan-1 under pathological conditions such as ischemia/reperfusion [44,76]. ROS is also the main factor inducing the degradation of HA [44]. The mechanism by which ROS directly damage the EGL is still unclear. A previous study showed that in a model of ischemia/reperfusion, xanthine-oxidoreductase (XOR) bound to the EGL via its heparin-binding domain (HBD), resulting in ROS generation in close proximity to the endothelial cell membrane, thereby disrupting the EGL integrity [77]. MPO induced the degradation of syndecan-1 probably via ionic interaction with heparan sulphate side chains, causing neutrophil-dependent syndecan-1 shedding and the collapse of the EGL structure [78].

## 3. Endothelial Glycocalyx Damage in COVID-19

Endothelial injury is considered as one of the important characteristics of severe COVID-19 patients with systemic inflammatory response syndrome, acute respiratory distress syndrome, microvascular thrombosis, Kawasaki disease, and multiple organ failure, all of which were closely associated with the extensive endothelial activation and dysfunction [2]. Numerous studies have observed the glycocalyx injury in severe COVID-19 patients [26,79,80]. The vascular endothelial glycocalyx damage caused vascular endothelial dysfunction, microvascular hyperpermeability, thrombosis, and leukocyte adhesion [33], and further aggravated the development of COVID-19 and delayed the recovery from vascular dysfunction [81]. 

### 3.1. Glycocalyx Fragments and Sheddases Are Elevated in COVID-19 Patients

The serum levels of HS, HA, and syndecan-1 are always used as the biomarker of EGL integrity and are detected in patients with sepsis, atherosclerosis, and diabetes [44]. The thickness of the EGL is also measured as an index of the layer injury in various experimental animals [43]. The circulating levels of glycocalyx fragments are now used to access the glycocalyx integrity and are believed to reflect the severity in COVID-19 patients [29,82]. Emerging evidence has showed that severe COVID-19 patients have higher syndecan-1 levels than healthy controls, and non-survivors have higher syndecan-1 levels than survivors of COVID-19 [28,82,83], which indicates that the high syndecan-1 level is associated with the increased mortality associated with COVID-19. HS is most abundant in lungs among all the mammalian organs and is ubiquitous through the alveol [81]. The higher plasma levels of HS and HA are also observed in severe COVID-19 patients [68]. The level and activity of heparanase, the exclusive mammalian HS-degrading enzyme, are elevated in COVID-19 patients [31]. Besides the circulating endothelial glycocalyx fragments, intravital microscopy was used to quantify the vascular density and EGL properties in sublingual microvessels and confirmed that the glycocalyx disruption contributes to the clinical progression of COVID-19 [26]. 

### 3.2. Endothelial Glycocalyx Damage Exacerbates ARDS in COVID-19

COVID-19 has caused an increase in ARDS and highlighted challenges associated with this syndrome. ARDS is characterized by pulmonary epithelial cell damage, severe inflammation, neutrophil adhesion or infiltration, and interstitial edema. The endothelial glycocalyx shedding plays an important role in the vascular endothelial hyperpermeability, adhesion, and neutrophil migration. Both endothelial barrier function and fluid clearance are weakened or inactive, which disturbed the EGL integrity, leading to pulmonary edema in ARDS [84]. HS has been evidenced to regulate the activation of the bradykinin pathway, which is involved in the local inflammation and vascular permeability [85]. Elevated plasma heparanase activity in COVID-19 patients can lead to the bradykinin pathway activation by the cleaving of HS and can subsequently trigger the inflammatory response and vascular leakage [85]. The heparanase-mediated EGL damage made an exposure of EC surface to adhesion molecules, facilitating the neutrophil adhesion and alveolar extravasation [81]. As a result, the EGL damage exacerbates the ARDS progression and delays the repair of endothelial injury in COVID-19 patients.

### 3.3. Endothelial Glycocalyx Damage Promotes Coagulopathy in COVID-19

Coagulopathy is one of the serious consequences of SARS-CoV-2 infection, leading to systemic coagulopathy similar to other serious infections, such as disseminated intravascular coagulation (DIC) [86]. Typically, the excessive activation of coagulation and pathological hyperfibrinolysis leads to disseminated intravascular coagulation (DIC), and the disruption of the glycocalyx contributes to this state. Clinical observation of 2000 patients found that the incidence of venous thromboembolism (VTE) in severe COVID-19 patients was as high as 35% [86]. Elevated D-dimer concentration, prolonged prothrombin time and slightly reduced platelet counts were observed in patients with COVID-19 [86,87]. Additionally, clinical studies showed that serum syndecan-1 was increased in response to the endothelial injury accompanied with the elevated D-dimer levels [29]. In previous studies, Iba [36] et al. proposed that syndecan-1 levels may not only relate to severity of sepsis but also to the development of DIC. Ikeda [88] et al. pointed that Syndecan-1 could be suggested as a predictor of DIC in sepsis, suggesting that syndecan-1 may be used as a predictive or diagnostic indicator of DIC in COVID-19. Together, under pathological conditions, the EGL will lose its protective effects from the endothelial surface, leading to DIC [89]. These findings revealed that reducing the risk of DIC in COVID-19 patients could be achieved by protecting the integrity of the EGL.

### 3.4. Multisystem Inflammatory Disease in Children and the Aged Associated with SARS-CoV-2

Kawasaki disease is characterized by systemic vasculitis of viral infection affecting children under 5 years of age. Recently, a multisystem inflammatory disease in children (MIS-C) associated with SARS-CoV-2 has been reported worldwide. MIS-C represents the severe inflammatory cytokine production and multi-organ dysfunction, sharing features with Kawasaki disease [90,91]. Previous studies showed that circulating endothelial glycocalyx components including syndecan-1 and HA were significantly elevated at the acute phase of Kawasaki disease and serum levels of syndecan-1 and HA were often applied to evaluate the vascular endothelial damage and the coronary artery lesions [92,93]. The case report of MIS-C showed elevated plasma syndecan-1 and pro-inflammatory cytokines, as well as the imaging biomarkers of endothelial glycocalyx degradation, including the total number of vessels, capillary recruitment, capillary density, and smaller diameter of venules [94]. In a recent clinical study, the endothelial glycocalyx degradation was observed in all MIS-C subjects, with elevated levels of syndecan-1 in blood and increased HS and CS in the urine. Moreover, the degree of the glycocalyx deterioration was highly corrected with disease severity [95]. More importantly, children have a unique endothelial surface layer that differs from adults, but studies evaluating glycocalyx damage in pediatric diseases are relatively few [96]. It is expectable to clarify the potential association between pediatric COVID-19 and Kawasaki disease, and further studies of glycocalyx biomarkers as the effective predictive indicators in children with MIS-C are warranted. 

It has been shown that in humans and mice, advanced age caused age-related deterioration of the glycocalyx, characterized by the thinning of the glycocalyx and altered glycocalyx barrier function [97]. In addition, aging is an important risk factor for severe COVID-19 patients [98]. COVID-19 mortality increased with age, accompanied with the decreased thickness of the glycocalyx [98,99]. Therefore, preventing the glycocalyx damage may be a new therapeutic direction for improving vascular dysfunction in elderly COVID-19 patients.

## 4. The Mechanisms of COVID-19-Induced Endothelial Glycocalyx Damage

As described above, SARS-CoV-2 infection-induced extensive endothelial insult and glycocalyx damage are closely linked to severe inflammation, thrombosis, and multi-organ failure in critically ill patients. The main factors involved in endothelial dysfunction as well as the involved signal pathways were summarized in Table 1. However, the molecular mechanisms underlying the glycocalyx injury caused by the virus remain largely unknown. According to the present studies, the inflammatory mediators, ROS, MMPs, the glycocalyx fragments, and viral proteins may contribute to endothelial glycocalyx damage in the pathogenesis of COVID-19.

### 4.1. The Pro-Inflammatory Cytokines and ROS-Induced Glycocalyx Degradation

The glycocalyx injury is inseparable from inflammatory response and oxidative stress. Inflammatory response and immune dysfunction caused by SARS-CoV-2 infection disrupted the redox homeostasis, resulting in the synthetic increase in ROS in immune or non-immune cells [111]. ROS and pro-inflammatory cytokines, such as IL-1β, IL-6, and TNF-α, activated sheddases including heparanase, MMPs, and hyaluronidase to induce glycocalyx degradation [112]. The positive feedback between activated sheddases and the cytokines further aggravated the inflammatory response and the EGL damage [117]. Clinical observation showed that patients with high levels of the pro-inflammatory cytokines also have high levels of glycocalyx degradation indicators, suggesting the relevance between glycocalyx damage and inflammation [26,27,28,29]. Likewise, increased plasma malondialdehyde (MDA) levels and ROS production in the fresh sputum of COVID-19 patients were detected [112]. The levels of circulating neutrophil extracellular traps (NETs) were elevated in COVID-19 patients, which indicated neutrophil activation and excessive ROS generation [118]. Potje et al. found that the treatment of human umbilical endothelial cells (HUVECs) with plasma from COVID-19 patients containing high levels of cytokines and MDA induced HS shedding and downregulated the protein levels of syndecan-1 and glypican-1 in vitro [112], indicating the involvement of cytokine- and ROS-induced glycocalyx degradation.

### 4.2. The Role of MMPs in the Degradation of Endothelial Glycocalyx

MMPs have been shown to mediate the shedding of syndecan-1 and HS [119]. The clinical study showed that MMP-1 and vascular endothelial growth factor A (VEGF-A) were significantly elevated in hospitalized COVID-19 patients when compared to mild/moderate cases [120]. In addition, excessively increased MMP-1 enzymatic activity is highly associated with the severe dysregulation of multiple EC activation markers in COVID-19 patients [120]. Another report showed that the plasma MMP-2 levels decreased while MMP-9 levels increased in severe COVID-19 patients, but the COVID-19 non-survivors had higher MMP-2 levels than COVID-19 survivors, which is considered to be related to the activation of the renin–angiotensin system [121]. Up to now, there are relatively few studies on the mechanism by which MMPs influence glycocalyx degradation. In a mouse model of type 1 diabetes, increased MMP activity was identified as a potential mechanism of endothelial glycocalyx damage, with loss of syndecan-4 [122]. In a mouse model of COVID-19, the administration of the ADAM17/MMP inhibitors significantly improved lung histology and prevented leukocyte infiltration and endothelial activation [123], suggesting its beneficial role in improving the endothelial function, although whether ADAM17/MMP inhibitors directly affect glycocalyx needs further investigation. A recent study pointed out that SARS-CoV-2 can induce genome-wide epigenetic changes in host cells, thus potentially affecting the expression of the MMPs through transcriptional, translational, and post-translational mechanisms [124]. It will be important to deepen our understanding of the role and molecular mechanism of MMPs in inducing glycocalyx degradation in severe COVID-19 patients.

### 4.3. Glycocalyx Fragments-Induced Endothelial Barrier Dysfunction

The density, glycosylation state, and length of the glycocalyx are considered the susceptibility factors of virus to host cell infection [25]. High levels of IL-1β, IL-6, and TNF-α in hyperinflammatory conditions, which are strong inducers of hyaluronidase, lead to the production and accumulation of HA in the alveolar spaces in severe COVID-19 patients [125]. The degraded glycocalyx fragments further interacted with endothelial cells and disrupted its barrier function [106]. HA fragments or low molecular weight (LMW) HA were reported to act as damage-associated molecular patterns (DAMPs) to activate the innate immune response [126]. Queisser [106] et al. observed the dysregulated HA biosynthesis and degradation in pulmonary microvascular ECs after SARS-CoV-2 infection, leading to the release of pathological HA fragments into circulation. The small HA fragments bound to their receptors CD44 and layilin to activate the Rho-associated protein kinase (ROCK) signaling pathway, which were known to disrupt the endothelial barrier integrity [106]. Hymecromone, a synthetic inhibitor of HA, has been shown to improve lymphopenia and lung lesions, promoting the recovery of clinical manifestations in SARS-CoV-2-infected patients [127]. These studies indicated the role for HA in the vascular pathology of COVID-19, and more components that induce endothelial dysfunction need to be further confirmed.

### 4.4. Viral Proteins May Be Involved in Glycocalyx Degradation

There is evidence that SARS-CoV-2 did not infect human endothelial cells in vitro without the over-expression of ACE2 [128], so the direct injury of endothelial cells by viral infection cannot explain the broad endothelial dysfunction observed in COVID-19 patients. In a previous study, Puerta-Guardo [37,129] et al. found that dengue virus non-structure protein 1 (NS1) induced the degradation of sialic acid and HS by upregulating of sialidases and heparanase, leading to hyperpermeability and disruption of the EGL. Moreover, dengue virus NS1-induced endothelial permeability and vascular leak can be prevented by NS1 vaccination [130]. These results implicated that the direct toxic effect of viral proteins may contribute to endothelial dysfunction and glycogalyx damage. Our previous work demonstrated that SARS-CoV-2 nucleocapsid protein is a specific endothelial activator that promotes endothelial inflammatory response and monocyte adhesion through TLR2-NF-κB/MAPK pathways [105]. The effect of SARS-CoV-2 nucleocapsid protein on endothelial dysfunction and glycocalyx damage is now ongoing in our lab and the potential molecular mechanism is also explored in vitro as well as in animal models, with the aim to further clarify the role of viral proteins in severe COVID-19 complications.

**Figure 2:**
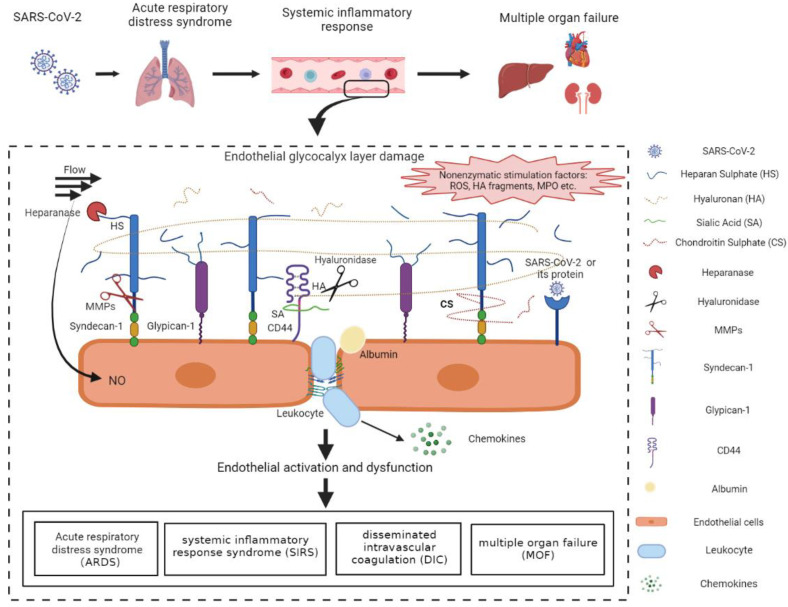
Mechanisms of the vascular endothelial glycocalyx damage in COVID-19 patients. SARS-CoV-2-infected lung epithelial cells activated the immune system and triggered the dysregulated host immune response in COVID-19 patients. The immune dysfunction disrupted the redox homeostasis, resulting in the excessive production of ROS. ROS and pro-inflammatory cytokines, such as IL-1β, IL-6, and TNF-α, were released into circulation and facilitated the activation of glycocalyx sheddases. The activated heparanase, hyaluronidase, and MMPs cleaved HS, HA, and sydecan-1, respectively, leading to the degradation of the EGL. The positive feedback between activated sheddases and the cytokines further aggravated the inflammatory response and the EGL damage. In addition, ROS, MPO, the degraded HA, as well as SARS-CoV-2 proteins may also induce the EGL disruption probably through the surface receptors and the downstream signal pathways. The endothelial injury, represented by endothelial activation and EGL perturbation, contributed mainly to the acute respiratory distress syndrome, systemic inflammatory response syndrome, disseminated intravascular coagulation, and multiple organ failure, which accelerated the progression of COVID-19 and increased mortality in COVID-19 patients. HA, hyaluronan; HS, heparan sulphate; CS, chondroitin sulphate; SA, sialic acid; MMPs, matrix metalloproteases; ROS, reactive oxygen species; MPO, myeloperoxygen; NO, nitric oxide. The figure was created by the online software tool BioRender.

Taken together, glycocalyx damage has been widely observed in severe COVID-19 patients and is regulated by multiple factors (Figure 2). However, the molecular mechanisms of direct glycocalyx damage induced by SARS-CoV-2 remain unclear and warrant further investigation. Medications to modulate the inflammatory response and MMP activity, suppression of gycocalyx degradation, and compounds that specifically target the viral proteins may be effective strategies to protect or restore the endothelial glycocalyx.

## 5. The Potential Therapy Targeting the EGL in COVID-19 Patients

Considering the roles of EGL in permeability, adhesion, and coagulation, repairing already damaged EGLs and preventing their further damage may be a promising therapeutic strategy for COVID-19 and its complications [131]. Up to now, however, the drugs used for COVID-19 treatment usually presented multi-functions and no agents that directly improve glycocalyx structure or integrity have been reported. Several treatment regimens that target the EGL repair and protection have been applied in clinical practice in COVID-19 patients (Table 2).

### 5.1. Heparin

The application of heparin significantly reduces COVID-19 mortality through protecting the damage of EGL and preventing the formation of thrombus [26,30,31]. Heparin has been widely reported as a heparanase inhibitor that plays an important role in sustaining EGL integrity [132,135]. In viral infections, heparin protected the vascular endothelial barrier via various manners including (1) the inhibition of heparanase activity, (2) interference with leukocyte trafficking, (3) neutralization of chemokines and cytokines, (4) inhibition of viral invasion [26,136], and (5) neutralization of extracellular cytotoxic histones [71]. In addition, the activation of heparanase upregulates the expression of MMP, so heparin may attenuate the increase in MMP expression through inhibiting heparanase activity [45,69].

### 5.2. Sulodexide

Sulodexide is a sulphated polysaccharide complex extracted from mammalian intestinal mucosa, consisting of HS (80%) and DS (20%) [137]. The biological activity of sulodexide comprises antithrombotic and profibrinolytic, anti-inflammatory, and beneficial hemorheologic effects [133]. Sulodexide has been shown to preserve endothelial glycocalyx function by increasing GAG synthesis and decreasing its catabolism [131]. Broekhuizen [138] et al. found that oral sulodexide administration improves EGL dimension, probably through enhancing the precursor abundance for GAG synthesis in patients with type 2 diabetes. The beneficial effects of sulodexide in COVID-19 patients were evidenced by numerous studies [133,139,140]. A randomized placebo-controlled outpatient trial showed that the patients in the sulodexide group had less hospitalization, less oxygen requirement, and lower D-dimer and CRP levels. In addition, treatment with sulodexide within 3 days of the onset could improve their clinical outcomes of COVID-19 patients [139]. Szolnoky considered that sulodexide may represent an alternative to heparin as a prophylactic agent in COVID-19 infections due to its sufficient intestinal absorption, safe use in renal insufficiency, and lower risk for heparin-induced thrombocytopenia, major bleeding, and drug-induced hypersensitivity [133].

### 5.3. The Corticosteroids

It is well known that corticosteroids (dexamethasone, hydrocortisone, methylprednisolone or equivalent, etc.) downregulated the native immune response with wide spectrum immunosuppression. It has been reported that the corticosteroids, particularly dexamethasone, exerted obvious therapeutic benefits in critically ill patients with COVID-19. Dexamethasone treatment has been proved to significantly reduce the mortality risk through multiple modulation including increasing the blood oxygen saturation, alleviating endothelial inflammation, avoidance of microcirculatory disturbances, and reducing the general pro-coagulative state [141,142]. The effects of corticosteroids on endothelial glycocalyx have been addressed recently. An animal study showed that dexamethasone treatment significantly inhibited MMP activity and reserved the expression of ZO-1 and syndecan-1 in a model of sepsis-induced vascular hyperpermeability [143]. The clinical study also proved that dexamethasone ameliorated endothelial injury and inflammation as evidenced by decreased plasma concentrations of Ang-2, ICAM-1, and syndecan-1 [144,145]. Nevertheless, few studies have identified the direct benefit of the corticosteroids on the EGL damage in COVID-19 patients. Corticosteroids as potential drugs targeting the glycocalyx in the treatment of COVID-19 should be further evaluated.

### 5.4. Tocilizumab

Tocilizumab, a IL-6R monoclonal antibody that prevents IL-6 binding and reduces IL-6 signaling, was mostly used in patients with the advanced stage of COVID-19 to prevent the cytokine storm [134]. Ikonomidis [32] et al. demonstrated that tocilizumab increased the glycocalyx thickness and improved endothelial function in rheumatoid arthritis patients through a profound reduction in inflammatory burden and oxidative stress. These results provided a novel mechanism for the effects of tocilizumab on COVID-19 patients [32]. The direct protection of tocilizumab against glycocalyx damage in COVID-19 patients should be further verified.

## 6. Summary

In summary, extensive endothelial activation and dysfunction is critically involved in the severe complications of COVID-19. Although endothelial activation and dysfunction-mediated inflammation and abnormal coagulation have been widely reported, mechanisms of direct glycocalyx damage induced by SARS-CoV-2 mostly remains unclear and warrants further investigation in animal models and clinic trails. Moreover, the therapeutic efficiency of drugs that protect the EGL should be confirmed in well-designed clinical trials and clinical practice. It is urgent to develop therapeutics that reduce inflammatory cytokine signaling and improve endothelial function in severe COVID-19 patients. Therefore, targeting these pathways is of great value to translate the present understanding of EGL damage to COVID-19 treatment.

## Figures and Tables

**Table 1:** Factors and signal pathways of endothelial dysfunction in the pathogenesis of COVID-19.

Factors	Signal Pathways	Effects in COVID-19
**Endothelial dysfunction**		
ACE2	Renin–angiotensin system and ACE-Angulation-II-AT1R	Endothelial dysfunction, organ damage, and clot formation [100,101]
Endogenous mitochondrial DNA	cGAS–STING pathway	[102]
SARS-CoV-2 nucleocapsid protein	Lectin pathway	Endothelial injury and microangiopathy [103]
**Endothelial activation**		
IL-1	TLR/NF-κB	Endothelial activation and damage [104]
SARS-CoV-2 nucleocapsid protein	TLR2-MAPK/NF-κB	Endothelial activation [105]
HS	Bradykinin pathway	Inflammatory response, EC-neutrophil adhesion, and vascular leakage [85]
**Barrier disruption**		
Hyaluronan	HA-CD44-ROCK	Barrier disruption [106]
SARS-CoV-2 spike protein	integrin αVβ3-VE-Cadherin	Barrier disruption [107]
S1 subunit of SARS-CoV-2 spike protein (S1SP)	-	Decreased microvascular transendothelial resistance and barrier function [108]
TNFα	Rho-kinase	Disruption of intercellular tight junctional proteins and the endothelial barrier [109]
Angiopoietin 2	Tie2 signaling	Increased endothelial permeability [110]
Heparanase	TNFα-heparanase-HS	Disruption of endothelial glycocalyx integrity, inflammation, and coagulation [111,112,113]
**Coagulation and thrombosis**		
Complement C5a	Complement pathway	Coagulation [114]
Complement C5b-9	Complement pathway	Endothelial activation and dysfunction and coagulation [114]
Complement C4d, MASP2, and C5b-9	Mannan-binding lectin (MBL) pathway	Endothelial necrosis and thrombosis [115]
PAI-1	STAT3	Coagulopathy and thrombosis [116]
Syndecan-1	-	Coagulation and endothelial injury [29]

MAPK, mitogen-activated protein kinases; NF-κB, nuclear factor NF-κB; TLR, Toll-like receptors; IL-1, interleukin-1; MASP2, mannose binding lectin (MBL)-associated serine protease 2; PAI-1, plasminogen activator inhibitor-1; STAT3, signal transducer and activator of transcription 3; ACE2, angiotensin-converting enzyme 2; AT1R, angiotensin II type 1 receptor; cGAS–STING, cyclic GMP-AMP synthase stimulator of interferon genes.

**Table 2:** Treatment related to endothelial glycocalyx in COVID-19 patients.

Drug	Potential Target in EGL	Effects Associated with Endothelial Function
Heparin	Inhibitor of heparanase	Protecting the vascular endothelial barrier [132]; reducing MMP expression [45,69]
Sulodexide	Increasing GAG synthesis and decreasing its catabolism [131]	Preserving endothelial glycocalyx function; antithrombotic, profibrinolytic, and anti-inflammatory effects [133]
The corticosteroids	Unknown	Inhibiting MMPs activity; reserving ZO-1 and syndecan-1 expression; reducing endothelial injury and inflammation [19]
Tocilizumab	IL-6R	Increasing the glycocalyx thickness; improving endothelial function [32,134]

## Data Availability

Not applicable.

## Abbreviations

EGL: endothelial glycocalyx layer; EC, endothelial cell; GAG, glycosaminoglycan; HA, hyaluronan; HS, heparan sulphate; CS, chondroitin sulphate; SA, sialic acid; MMPs, matrix metalloproteases; ARDS, acute respiratory distress syndrome; DIC, disseminated intravascular coagulation; MIS-C, multisystem inflammatory syndrome; MOF, multiple organ failure; NO, nitric oxide; ROS, reactive oxygen species; MDA, malondialdehyde; HUVEC, human umbilical endothelial cells; ACE2, angiotensin-converting enzyme 2.
